# Neutron Scattering Study of a Frustrated Kagome-Strip Structure: Na_2_Co_3_(AsO_4_)_2_(OH)_2_

**DOI:** 10.1063/4.0000817

**Published:** 2025-10-27

**Authors:** Emily D Williams, Duminda Sanjeewa, Stuart Calder, Tianran Chen

**Affiliations:** 1 Clemson University, Clemson, South Carolina, USA; 2 Oak Ridge National Labratory, Oak Ridge, Tennessee, USA; 3 University of Tennessee, Knoxville, Tennessee, USA

## Abstract

The Na2Co3(AsO4)2(OH)2 structure has garnered interest owing to its mix of two-dimensional honeycomb and Kagome lattice features to form a Kagome-strip motif of S = 3/2 high-spin Co2+ metal centers. The Kagome-strip is believed to possess the same magnetically frustrated interactions as its parent honeycomb and Kagome lattices, both of which are well-known for their high magnetic frustration potential. Single crystals of Na2Co3(AsO4)2(OH)2 were prepared via a hydrothermal method. The pink columnar crystals crystallize in C2/m with cell parameters of a = 14.5885(9), b = 5.9376(3), and c = 5.0992(3) Å with β = 103.63(2)°. Magnetization studies show antiferromagnetic ordering at 14 K that is suppressed through a metamagnetic antiferro- to ferromagnetic transition that occurs at applied fields greater than 40 kOe, as revealed by isothermal field-dependent magnetization studies below 14 K. Magnetic structure determination by powder neutron diffraction yielded a k-vector of (0.5,0.5,0.5), confirming the antiferromagnetic behavior in low-field magnetization.

Both Co(1) and Co(2) moments lie in the bc-plane. Specifically, the Co(1) moments point along the c-axis, while the Co(2) moments rest along the b-axis. Magnetic studies presented herein suggest a highly frustrated system with complimentary powder neutron diffraction data providing a pathway for additional transition metal arsenate and vanadate analogs each with their own unique magnetic interactions owing to a difference in the transition metals themselves and in the interaction pathways afforded through p- and d-orbitals in the respective arsenate and vanadates.

